# The diving katydid: A unique predator escape behavior in *Ragoniella pulchella* (Orthoptera: Tettigoniidae)

**DOI:** 10.1002/ecy.70466

**Published:** 2026-07-21

**Authors:** Charlie Woodrow, Benjamin C. Bluck, Fabio Sarria‐S, Lewis B. Holmes, Juan Sebastián Ulloa, Fernando Montealegre‐Z

**Affiliations:** ^1^ School of Natural Sciences, Joseph Banks Laboratories University of Lincoln Lincoln UK; ^2^ Department of Ecology and Genetics Uppsala University Uppsala Sweden; ^3^ International Centre for Ecohydraulics Research, Faculty of Engineering and Physical Sciences, Boldrewood Innovation Campus University of Southampton Southampton UK; ^4^ School of Ocean and Earth Science, Faculty of Environment and Life Sciences University of Southampton Southampton UK; ^5^ Instituto de Investigación de Recursos Biológicos Alexander von Humboldt Bogotá D.C. Colombia

**Keywords:** bromeliad, bush‐cricket, natural selection, predation, semi‐squatic

Since their colonization of land over 400 million years ago, insects have made several transitory and permanent returns to aquatic environments. In particular, many adult forms have evolved specialized diving behaviors for transitory use of water and possess unique traits for enhancing dive duration, such as external connections to the air and physical gills. These behaviors have various purposes, including predator and parasitoid avoidance (Calosi et al., 2007), foraging (Hounslow et al., 2022), and thermoregulation (Kooyman, 2005). As such, diving has independently evolved across many major insect lineages. One insect order with a diversity of semiaquatic behaviors is the Orthoptera. These range from simple water associations in many taxa (Song, 2019) to hind leg adaptations for swimming in Acrididae (Carbonell, 1957) and to true diving in Tetrigidae (Amédégnato & Devriese, 2008), Acrididae (Gudowska et al., 2016), and Anostostomatidae (Derka & Fedor, 2010; Issa & Jaffe, 1999). In adults of the genus *Hydrolutos* Issa & Jaffe, 1999 (Anostostomatidae), a plastron‐like adaptation on the pleuro‐sternal area of the thorax and abdomen allows for diving times of up to 20 minutes (Derka & Fedor, 2010; Issa & Jaffe, 1999). It has also been suggested that the extinct Mesozoic family Elcanidae used flattened spurs of the hind tibiae for evasive locomotion in water like modern Tridactylidae (Burrows & Sutton, 2012; Chopard & Callan, 1956; Kim et al., 2021), suggesting a long evolutionary history of semiaquatic associations in Orthoptera. However, in katydids (Tettigoniidae) and crickets (Gryllidae), few interactions with water for swimming or diving behaviors have been identified (Song, 2019).

During recent field work (2022–2024) to the cloud forests of Valle del Cauca, Colombia, we discovered a novel antipredator diving behavior in *Ragoniella pulchella* (Orthoptera: Tettigoniidae: Conocephalinae) Hebard 1927, a tropical katydid reported initially from Boyacá, Colombia, and subsequently in the cloud forest of the Western Cordillera (Chamorro‐Rengifo et al., 2011, 2015; Montealegre‐Z, 1997). This species is found to inhabit large tropical bromeliads (Figure 1A) and was observed to dive into the central bromeliad pool in response to vibratory cues. Primarily, we observed this behavior in response to the presence of bromeliad‐dwelling spiders, but a similar response could be elicited by the observer tapping the bromeliad leaf. To formally describe this behavior, we conducted simple experiments in the lab and field to understand dive duration and identify potential oxygen‐carrying mechanisms and temperature sensitivity of dive duration. For measurements in the field, our data collection involved recording dive duration by artificially eliciting escape dives into bromeliads. Individuals of *R. pulchella* were typically observed positioned with their head downwards (Figure 1B), toward the bromeliad pools, with males sometimes singing in this position. Along the anterior–posterior axis of the animal in this position, their coloration matches that of the bromeliad, with the head and front of the body brown (similar in color to the water tank in the bromeliads), while the tegmina are green (similar to the leaves), which could be a form of camouflage (Figure 1B,C). This would suggest the observed coloration is a diurnal adaptation. When the vibratory stimulus of a gentle tap on the tip of the bromeliad leaf is presented in the field, *R. pulchella* runs or jumps down into the bromeliad pool (Video S1). In our field tests, individuals remained submerged for 112.6 ± 86.5 s (Appendix S1: Figure S1). Although singing males were more often observed in the central bromeliad tank, this behavior and dive duration did not differ across sexes (generalized linear model [GLM], *F* = 0.93, df = 29, *p* = 0.41, *N* = 5 males, 2 females). Once in the water, the respiratory spiracles close. As the insect sits in the water, there is a visible pumping of the body, which causes a substantial bubble of air to expand and contract from the prothoracic (acoustic) spiracle, the large opening to the acoustic trachea (ear canal) of the insect (Figure 1C, Video S2). This likely comes from expansions and contractions of the respiratory system, from which the acoustic system is derived and remains internally coupled. Toward the end of the diving behavior, individuals walk backwards out of the water, often first only extending the distal few abdominal segments above the surface before fully leaving the water.

**FIGURE 1:**
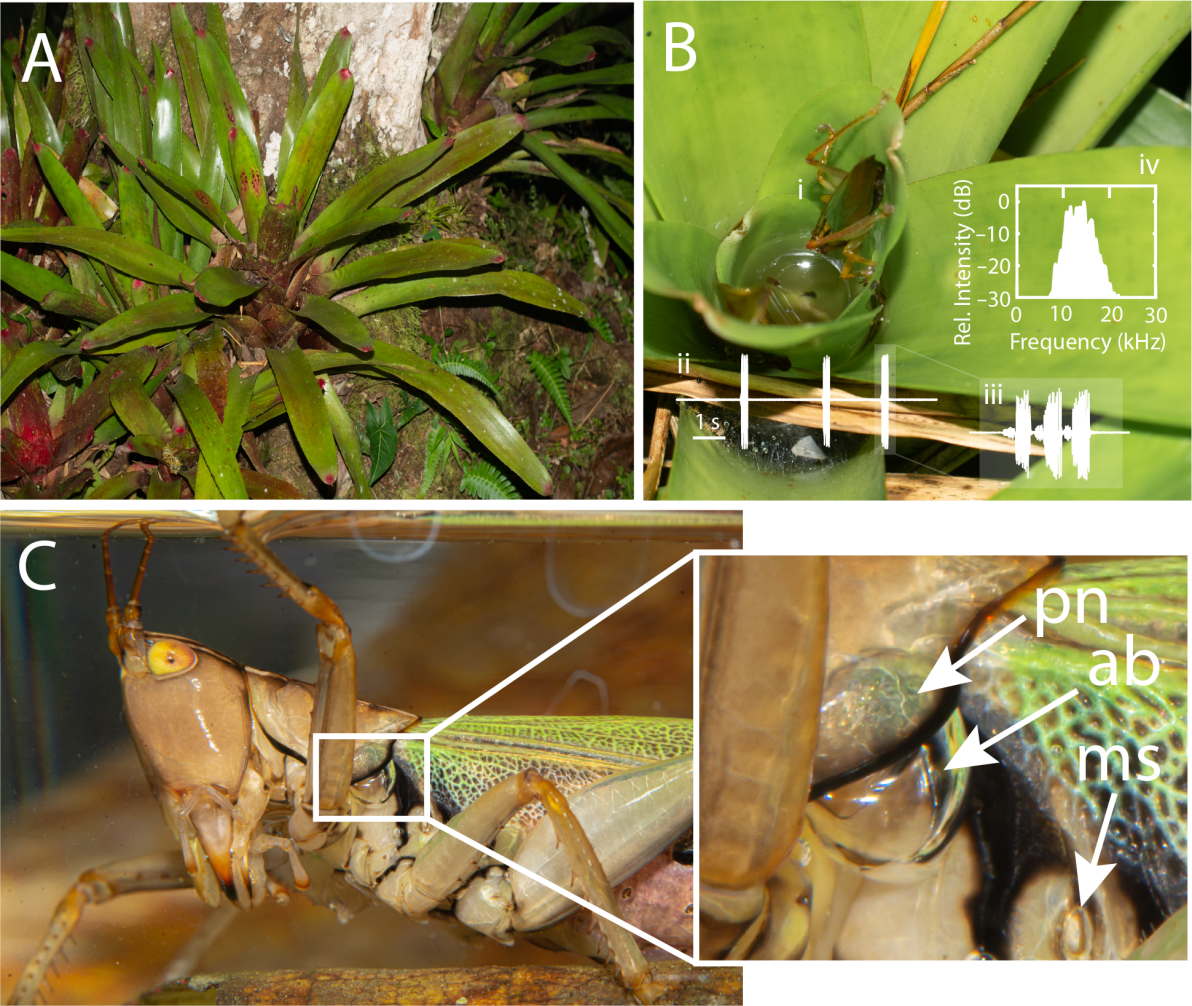
Bromeliad and water associations in *Ragoniella pulchella* Hebard 1927. (A) Bromeliad environment where the species is typically found. (B) Singing position of *R. pulchella* (i), with example song repertoire (ii), detail of a single chirp (iii), and frequency spectrum (iv). (C) Submerged *R. pulchella* with visible air bubble (ab) at the prothoracic spiracle supported by an inflation of the pronotal lobe (pn). Conversely, the mesothoracic spiracle (ms) is closed. Photos by Charlie Woodrow.

Following field observations, we simulated a predator approach in a controlled laboratory setting to test the effect of water temperature and volume on dive duration. We hypothesized that increased water temperatures would decrease dive time, likely due to increased use of oxygen through increased metabolic rate. We also tested different water volumes as we hypothesized that at small water volumes, the water tank may be perceived as not providing a beneficial antipredator benefit (the insect may not be able to submerge the body), and thus, dive time would be reduced. Additionally, if gas transfer through the air bubble was occurring, gas transfer would be reduced when the water volume is reduced, which could also reduce dive times. A 1‐L bottle of water was placed into a temperature‐controlled incubator (PHCBI MIR‐154) for 24 h to simulate sitting water in a bromeliad after rainfall. We tested a temperature range of 16–26°C, at 2°C intervals, covering the range of temperatures this species would experience in nature. In a 250‐mL glass beaker, a volume of either 100, 150, or 200 mL of water was added, and a real bromeliad leaf (*Guzmania* sp.) was placed semi‐submerged as an initial resting place for the insect. To initiate diving behavior, the tip of the bromeliad leaf was suddenly tapped by the experimenter, causing the katydid to run down the leaf into the water. The time to total submergence was almost immediate (<1 s), and did not show any clear variation. Therefore, the trial was considered started when the specimen was fully submerged under the water and stable (i.e., able to grip onto a surface). The stopwatch was stopped, and the trial was considered finished as soon as any part of the body with respiratory spiracles was clear of the water surface. Body length (frons to last tergite) was used as a proxy of body size and was measured after the laboratory experiments were completed. We observed that dive duration was around 179 s longer in the lab than in the field, averaging 292 ± 233.9 s (Appendix S1: Figure S1; GLM, *N* = 180 dives, *F* = 17.1, df = 209, *p* < 0.001). We also found that dive times are reduced when the water is warmer (Figure 2A; *F* = 75.57, df = 179, *p* < 0.001), which is likely due to increased metabolic rate and thus more rapid use of stored oxygen at higher temperatures (Davies & Tribe, 1969; Fry & Hart, 1946; Fuhrman & Fuhrman, 1959), but could also be due to reduced dissolved oxygen in warmer water, if the air bubble is being used as a physical gill. Interestingly, we also observed that dive duration was dependent on the volume of water (Figure 2D; GLM, *F* = 3.88, df = 179, *p* = 0.022). Pairwise contrasts revealed that this effect was due to significantly lower dive durations for 100 mL compared to 200 mL (estimate = −70.3 s, df = 173, *t*‐ratio = −2.620, *p* = 0.026). If *R. pulchella* can detect that the water volume is not sufficient to hide from a predator, they may switch to other more typical escape behaviors such as flight or jumping. This was clear in our experiments as dives were often aborted at water volumes of 100 mL, and the individuals would jump frantically after leaving the water which we did not observe for other water volumes. Such an issue may have been compounded by the experiments being conducted in transparent beakers, and the researchers thus being visible to test individuals, so further studies are needed. In a bromeliad, potential predators would not be visible, or only visible in one specific direction, and it is likely the katydids would instead rely more heavily on vibrational cues. This could also explain the increased dive durations in the lab experiments compared to the field which were conducted with red light, which most insects cannot perceive (Briscoe & Chittka, 2001). It is also possible that this change is behaviorally adaptive instead. If *R. pulchella* and their main predators are primarily nocturnal, they may associate the colder night temperatures of both air and water with increased predation risk. This may accordingly lead them to spend longer underwater if they perceive the relative risk to be higher. Further experiments are required to understand this behavior in different predation contexts, and also to understand how long the predator remains present while the insect is submerged.

**FIGURE 2:**
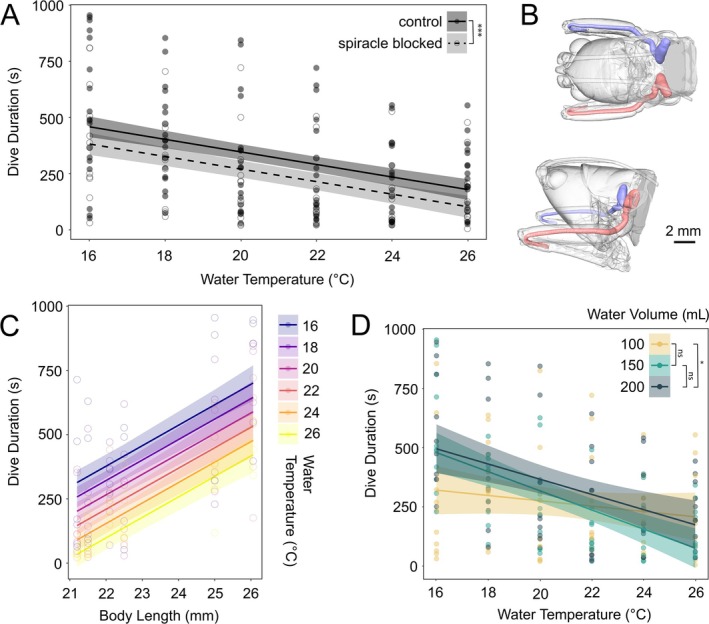
Dive duration is affected by water temperature, body size, water volume, and the availability of stored oxygen. (A) Effect of water temperature on dive duration for control experiments and spiracle blocking experiments. (B) Acoustic tracheae of *Ragoniella pulchella* in dorsal and lateral view to highlight the volume of stored oxygen. (C) Effect of body length on dive duration at different water temperatures. (D) Effect of water volume on dive duration. All lines represent predicted values from fitted models and shaded areas represent 95% CIs. For fixed effects not shown in each panel (e.g., panel A does not show body length, but it is included in the model), average values were used to obtain model fits. ns, not significant, *p < 0.05, **p < 0.01, ***p < 0.001﻿﻿﻿﻿﻿﻿. Micro‐CT images by Charlie Woodrow.

Finally, to try and understand whether *R. pulchella* uses the pumping of air from the large acoustic trachea as a specialized mechanism for oxygen carrying/extraction, we blocked the acoustic spiracles with a fast‐drying non‐toxic latex, to test if dive times were reduced when the air bubble pumping observed in Video S2 is prevented. Dive times were significantly reduced when the acoustic spiracles were blocked (Figure 2A; estimate = −76 s, *F* = 11.58, df = 179, *p* < 0.001). As with the field experiments, our lab experiments found that sex had no effect on dive duration (*F* = 0.51, df = 179, *p* = 0.47), with the observed differences in dive time between males and females instead being explained by differences in body size (*F* = 187.03, df = 179, *p* < 0.001), whereby larger individuals have longer dive durations (Figure 2C). μ‐CT scanning (SkyScan 1172 μ‐CT scanner, Bruker Corporation, Billerica, MA, USA, voxel size 9 μm, voltage 55 kV, current 180 mA, exposure 200 ms, rotation step 0.2°) revealed that the volumes of air held by the acoustic trachea averaged 2.85 ± 0.42 mm^3^ (Figure 2B; *n* = 3 male, 4 female), and the area of the opening of the acoustic spiracle averaged 0.69 ± 0.04 mm^2^ (Appendix S1: Table S1). Together, the large volume of air held by the acoustic tracheae, the observed pumping bubble (Video S2), and the reduced dive time when this behavior is prevented, indicate a potential role of the acoustic system as a specialized oxygen retention/acquisition mechanism. As this trachea is internally coupled to the respiratory system through small tracheoles, the benefit of the acoustic system on dive duration may simply be an increase in the volume of air the insect can carry. A more detailed anatomical study of the tracheal system in *R. pulchella* (requiring higher resolution 3D imaging) would be beneficial for understanding whether the trachea serves a more sophisticated oxygen‐carrying function. It is also important to note that we were not able to include the latex on another part of the insect for a control group in this study. While no apparent adverse response to the latex was observed, we nonetheless cannot rule out contributions of other physiological effects the latex may have on the insect. If the blocking of the spiracle has reduced dive time through other effects, it may be that the role of the air bubble is not for enhanced dive time, but to prevent water entering the hearing system so that on emergence from the water, the insect can listen to its environment immediately. This could be tested by removing the pronotum extension observed in Figure 1C in a live or dead specimen, to see if the bubble is still held.

While further observations of the range of predators which induce this behavior are required, we observed an abundance of semiaquatic spider species cohabiting the bromeliads of *R. pulchella*. These spiders, particularly of the genus *Cupiennius*, were regularly observed predating on katydids and other Polyneoptera during field observations (Appendix S1: Figure S2). Their semiaquatic young were observed partially or fully submerged in the central tank, while the larger individuals which predate katydids are too large to fit in, or reach into, the central bromeliad tank, which therefore acts as a refuge for *R. pulchella*. Among other invertebrate and vertebrate species, predator avoidance/defense is a common use of bromeliad tanks (de Oliveira Souza et al., 2024; Romero et al., 2007). For example, the frog genus *Crossodactylodes* are largely bromeliad‐dwelling, spending their whole lives in and around bromeliads. *Crossodactylodes* frogs are also bromeligenous, laying and raising their young in bromeliads. Observations of the species *Crossodactylodes itambe* have noted that while inactive and unthreatened, they rest near the bromeliad's inner water line. However, when threatened, their escape behavior involves jumping into the water and enacting a fast dive deeper into the tank water (Barata et al., 2018). Tadpoles inhabit the tank water for the significant majority of their development and also enact similar diving behavior when threatened (Santos et al., 2017). Similar escape diving behaviors have also been observed in the Trechaleid spider *Cupiennius salei* (Hénaut et al., 2018). Among insects, various species of mosquito and damselfly are also bromeligenous (Frank et al., 1981; Srivastava et al., 2020), using bromeliad tanks to rear young through the aquatic stages of their development. Notably, the predatory larvae of the damselfly *Mecistogaster modesta* occur almost exclusively in tank bromeliads of water volume >100 mL, seemingly due to the comparatively reduced risk of desiccation in larger tanks (Srivastava et al., 2020). While within the water tanks, the predatory larvae will feed on other invertebrates, such as other larvae and beetles. Thus far in Orthoptera, only nymphs of the cone‐headed katydids (Conocephalinae, the same subfamily as *R. pulchella*) have been observed to interact with bromeliad tanks, sitting partially submerged to ambush prey (Burmeister, 1985), rather than to escape their own predators as in *R. pulchella*.

The bromeliads which *R. pulchella* inhabits are central tank types, in which collected rainwater sits within the central, apical tank of the plant. This type of bromeliad has been demonstrated to be much more efficient at maintaining moisture than other bromeliad tank types (Zotz & Thomas, 1999), which may explain the preference of *R. pulchella* for this habitat, particularly during the dry season. In addition, it has been shown that larger tanks are better at maintaining moisture (Srivastava et al., 2020; Zotz & Thomas, 1999), which, combined with the benefit of a larger water volume for increased dive times and thus improved predator avoidance, supports observations that bromeliads are an important resource for this species. Little is known about whether the bromeliads form other important functions in *R. pulchella*, such as habitat for specialized prey foraging (Burmeister, 1985), oviposition sites (Frank, 1986; Osses et al., 2008; Saul‐gershenz, 1993), or elevated signaling perches (Hall & Robinson, 2021; Hartmann et al., 2004). However, they are commonly occupied by other neotropical katydid species (Lang & Römer, 2008; Montealegre‐Z & Morris, 1999), indicating they serve several functions. In the larger environments where specimens were kept before and after experiments, one female was observed ovipositing between the leaves of a bromeliad, but more observations and a better understanding of the life history of this species are required.

Diving behaviors are common in insects but rare in crickets, katydids, and allies. This novel diving behavior expands upon our understanding of the aquatic behaviors of terrestrial invertebrates and highlights the diversity of potential oxygen retention mechanisms in diving insects. Further research to test for this behavior in the field across other neotropical species, assess the metabolic costs of diving, and measure how predator vibrational cues are transmitted across bromeliads could provide key insights into the evolution of this unique predator avoidance strategy.

## CONFLICT OF INTEREST STATEMENT

The authors declare no conflicts of interest.

## Supporting information


Appendix S1.

Video S1.

Video S1_Metadata.

Video S2.

Video S2_Metadata.

## Data Availability

Data (Woodrow, 2026) are available in Figshare at https://doi.org/10.6084/m9.figshare.32114563.v3.
