# Supplementary material for: The diving katydid: A unique predator escape behavior in Ragoniella pulchella (Orthoptera: Tettigoniidae)
Source: Ecology. 2026 Jul 21;107(7):e70466. doi: 10.1002/ecy.70466 (PMC13389546; doi:10.1002/ecy.70466)
Supplement: Supplementary file 1 — Appendix S1. [file ECY-107-e70466-s001.pdf]

Appendix S1

The diving katydid: A unique predator escape behavior in *Ragoniella pulchella* (Orthoptera: Tettigoniidae)

Charlie Woodrow, Benjamin C. Bluck, Fabio Sarria-S, Lewis B. Holmes, Juan Sebastián Ulloa, Fernando Montealegre-Z

Ecology

Table S1. Morphological measurements of specimens used for μ-CT scanning. For paired features, both the left and right measurements are provided (L/R). na = not applicable (feature missing).

Individual ID	Sex	Body length (mm)	Trachea length (mm)	Trachea volume (mm^3)	Acoustic spiracle area (mm^2)
FMZa33	F	31.16	18.06/na	3.19/na	0.71/na
FMZa11	M	19.80	17.42/17.68	2.80/2.85	0.70/0.73
FMZa49	F	13.80	18.53/na	3.75/na	0.75/na
F1	F	26.06	19.56/19.17	3.22/3.21	0.75/0.70
M1	M	25.00	18.31/18.58	2.31/2.79	0.69/0.70
M3	M	22.50	17.25/17.60	2.24/2.76	0.66/0.64
M4	M	21.70	17.34/16.40	2.54/2.57	0.65/0.66

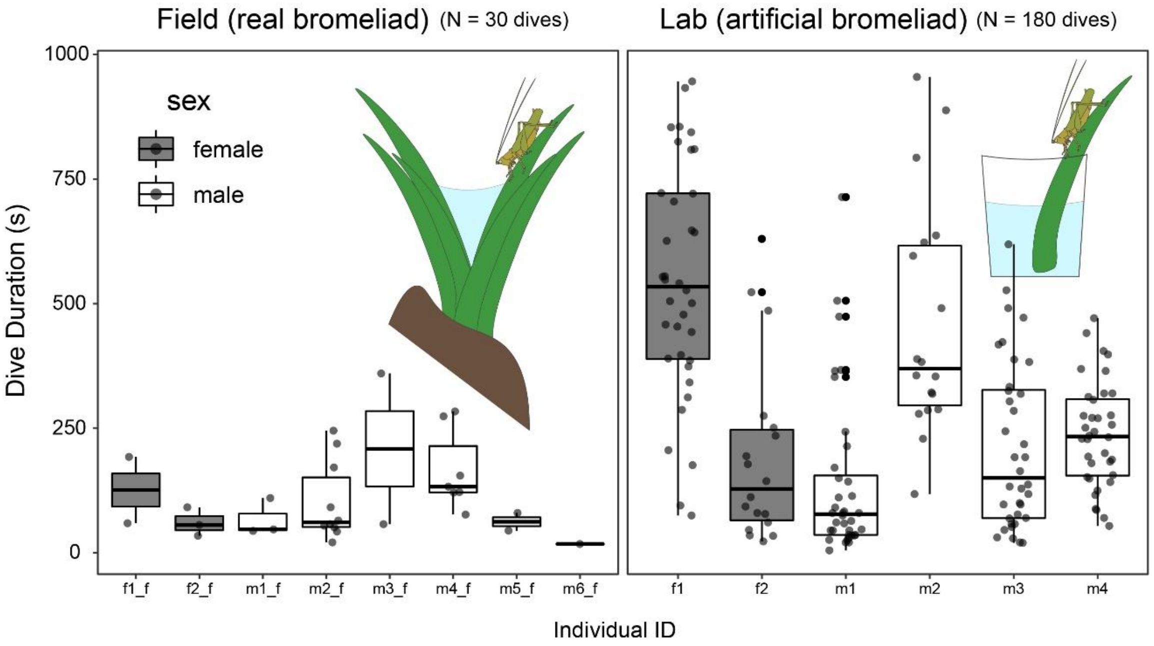

Figure S1. Dive durations of each individual measured in the lab and field. All measured individuals were adults except *m6_f*, which was a male nymph (~5th instar). Illustrations not to scale. Illustrations by Charlie Woodrow.

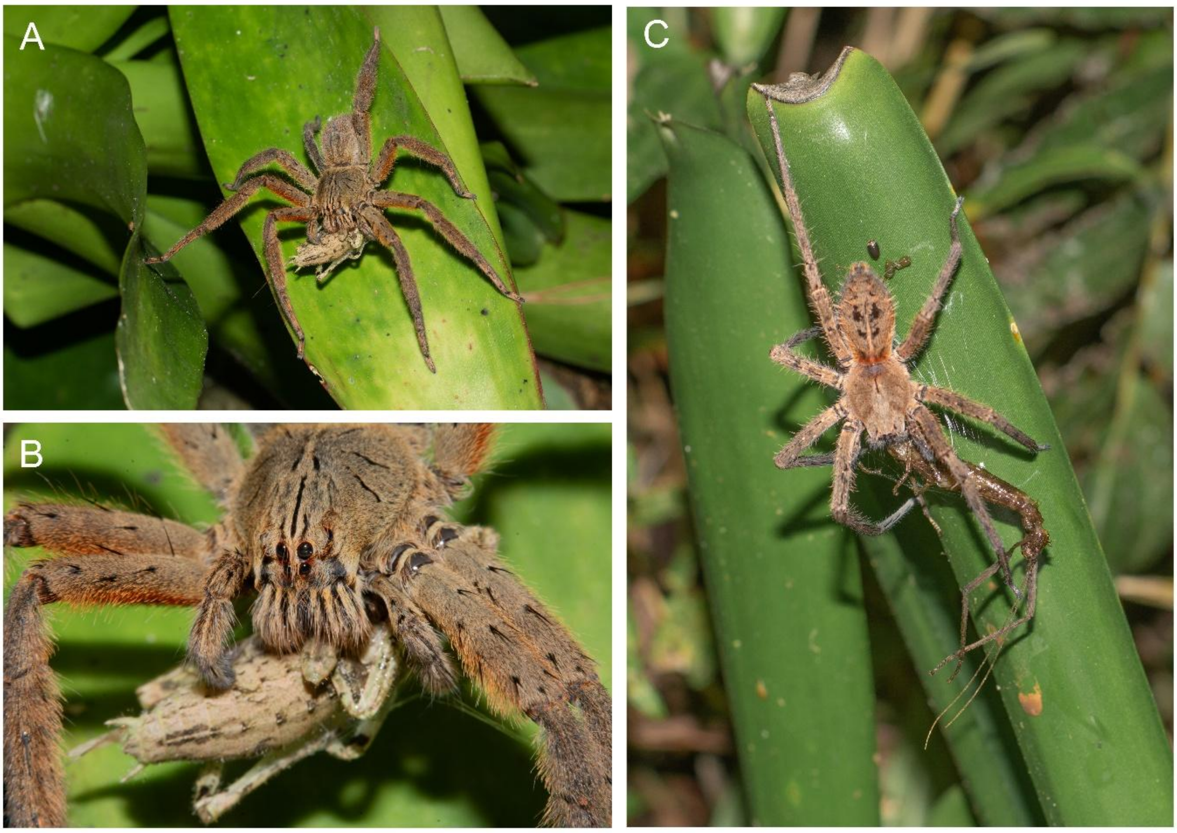

Figure S2. Bromeliad inhabiting spiders observed to be predating Orthoptera and allies. (A) red-legged wandering spider (*Cupiennius coccineus*) predating Pseudophylline katydid. (B) close up view of A. (C) bromeliad spider (*Cupiennius sp.*) predating a phasmid. Photos by Charlie Woodrow.
