# Supplementary material for: The diving katydid: A unique predator escape behavior in Ragoniella pulchella (Orthoptera: Tettigoniidae)
Source: Ecology. 2026 Jul 21;107(7):e70466. doi: 10.1002/ecy.70466 (PMC13389546; doi:10.1002/ecy.70466)
Supplement: Supplementary file 3 — Video S1_Metadata. [file ECY-107-e70466-s005.pdf]

Video S1 Metadata

The diving katydid: A unique predator escape behavior in *Ragoniella pulchella* (Orthoptera: Tettigoniidae)

Charlie Woodrow, Benjamin C. Bluck, Fabio Sarria-S, Lewis B. Holmes, Juan Sebastián Ulloa, and Fernando Montealegre-Z

Ecology

Video S1. Induced predator escape diving by *R. pulchella* in response to artificial vibratory stimulus. Videographer credit: Charlie Woodrow.
