# Supplementary material for: The diving katydid: A unique predator escape behavior in Ragoniella pulchella (Orthoptera: Tettigoniidae)
Source: Ecology. 2026 Jul 21;107(7):e70466. doi: 10.1002/ecy.70466 (PMC13389546; doi:10.1002/ecy.70466)
Supplement: Supplementary file 5 — Video S2_Metadata. [file ECY-107-e70466-s003.pdf]

Video S2 Metadata

The diving katydid: A unique predator escape behavior in *Ragoniella pulchella* (Orthoptera: Tettigoniidae)

Charlie Woodrow, Benjamin C. Bluck, Fabio Sarria-S, Lewis B. Holmes, Juan Sebastián Ulloa, and Fernando Montealegre-Z

Ecology

Video S2. Video of recycled air bubbles being displaced from the acoustic trachea during diving in *R. pulchella*. This air should theoretically be available for the neighboring respiratory spiracle which the expanding bubble engulfs. Videographer credit: Charlie Woodrow.
